# Fast and antibiotic free genome integration into *Escherichia coli* chromosome

**DOI:** 10.1038/s41598-020-73348-x

**Published:** 2020-10-05

**Authors:** Esther Egger, Christopher Tauer, Monika Cserjan-Puschmann, Reingard Grabherr, Gerald Striedner

**Affiliations:** 1 Christian Doppler Laboratory for Production of Next-Level Biopharmaceuticals in E. Coli, Institute of Bioprocess Science and Engineering, Department of Biotechnology, University of Natural Resources and Life Sciences, Muthgasse 18, 1190 Vienna, Austria; 2 Austrian Centre of Industrial Biotechnology, Muthgasse 11, 1190 Vienna, Austria

**Keywords:** Biotechnology, Molecular biology

## Abstract

Genome-based *Escherichia coli* expression systems are superior to conventional plasmid-based systems as the metabolic load triggered by recombinant compounds is significantly reduced. The efficiency of T7-based transcription compensates for low gene dosage (single copy) and facilitates high product formation rates. While common Gene Bridges’ λ-red mediated recombination technique for site directed integration of genes into the host genome is very efficient, selection for positive clones is based on antibiotic resistance markers and removal thereof is often time consuming. For the generation of industrial production strains, flexibility in terms of integration site is not required, yet time from gene design to a stable clone is a quite relevant parameter. In this study, we developed a fast, efficient and antibiotic-free integration method for *E. coli* as production strain. We combined the λ-red recombination system with the site-directed homing endonuclease I from *Saccharaomyces cerevisiae* (I-SceI) for selection. In a first step, λ-red proteins are performing genome integration of a linear, antibiotic marker-free integration cassette. The engineered host strain carries the I-SceI restriction sequence at the attTn7 site, where the integration event happens. After homologous recombination and integration at the target site, site-specific genome cleavage by endonuclease I-SceI is induced, thereby killing all cells still containing an intact I-SceI site. In case of positive recombination events, the genomic I-SceI site is deleted and cleavage is no longer possible. Since plasmids are designed to contain another I-SceI restriction site they are destroyed by self-cleavage, a procedure replacing the time-consuming plasmid curing. The new plasmid-based “All-In-One” genome integration method facilitates significantly accelerated generation of genome-integrated production strains in 4 steps.

## Introduction

*E. coli* constitutes an attractive, widely used and well characterized organism for recombinant protein production. The efficiency and convenience of its application in bioprocessing and manufacturing of biopharmaceuticals are only a few of many advantages of this bacterial system^1, 2^. Huge varieties of host/vector systems especially based on various promoters and on different cultivation and induction strategies exist and have been applied in the past^3^. Striedner et al.^4^ showed that genome-integrated T7-based expression systems, in which the target gene is site-specifically integrated into the host, are superior to conventional plasmid-based systems. For genome integration of expression cassettes, the attTn7 site is highly attractive for recombinant protein expression without negative influence on the hosts metabolism^4, 5^. Naturally, the bacterial transposon attTN7 integrates at this site, upstream of the *glmUS* operon, which encodes two proteins involved in cell wall biosynthesis^6^. In genome integrated systems, the metabolic load triggered by recombinantly produced compounds is significantly reduced, whereby plasmid-loss, which is a common source of instability for conventional expression systems, is eliminated. Also, by providing a lower gene dosage as compared to high copy plasmid-based expression, leakiness of the T7-system can be minimized. The efficiency of the T7-RNA polymerase compensates for the single gene copy and facilitates high product formation rates without fatal consequences to the host metabolism.

Thus, for stable and robust recombinant protein expression in *E. coli*, integration of the target gene into the genome is preferable. Therefore, various strategies have been established and are readily available^7^. Apart from the variations of the recombination enzymes used, these methods differ in their flexibility of integration site, in copy number and optimal length of integration cassettes, as well as scar-forming or scarless integration of target genes. One widely used and convenient method is the λ-red system. It offers an effective way of performing homologous recombination, where interaction of the λ-phage recombination genes *gam*, *bet* and *exo* leads to increased recombination events of linear DNA at the desired target site^8^. Following the protocol of Sharan et al.^9^, integration of double stranded DNA fragments flanked by 50 base homologies can easily and reproducibly be performed. Homologous flanking regions are simply added by primer-overhangs to the target gene or gene cassette. For selection of successful integration, an antibiotic resistance marker gene needs to be co-integrated. However, during a recombinant production process, the expression of an antibiotic resistance gene or any other selection marker is unwanted, since this means an additional metabolic burden for the cell which often results in a loss of the product. Therefore, after selecting the desired mutants, this marker has to be deleted by the action of the recombination enzyme flippase^10^. Following this protocol, construction of a genome integrated clone, free from antibiotic resistance marker and helper plasmid, takes at least 2 weeks, whereas FRT-scars still remain in the genome. For marker-less genome integration and for integration of multiple genes of interests (GOI), or multiple copies of one GOI, techniques have been developed that often combine the λ-red system with the action of the homing endonuclease I-SceI^11–16^. This restriction enzyme originally initiates double strand breaks in the *Saccharomyces cerevisiae* genome and is responsible for intron mobility in mitochondria of yeast^17^. The I-SceI-recognition site consists of an 18 base pair non-palindromic sequence which does not naturally occur in the *E. coli* genome. In these integration protocols, I-SceI is used for excision of target gene cassettes from a donor backbone, releasing them for integration at the chosen target site. Alternatively, the λ-red system is used in combination with the CRISPR/Cas9 system, which is highly efficient for selection of successful genome integration^18–22^. The RNA-directed endonuclease Cas9 can be guided to nearly every genomic site, where it cuts the DNA and thereby kills the cell. The requirement for Cas9 induced restriction is the existence of a protospacer adjacent motif (PAM) close to the target site. In case of positive integration, the original DNA-sequence is altered, homology to crRNA disappears and genome cutting is prohibited. Such systems consist of two different components: the CRISPR-associated (Cas) protein and a guide RNA consisting of a trans-activating CRISPR RNA (tracrRNA) and a programmable CRISPR targeting RNA (crRNA)^23^. The great flexibility concerning DNA restriction sites is certainly the major advantage of the CRISPR/Cas9 system although, off target effects and the requirement for PAM must be considered. However, in many industrial applications the production strain as well as the expression site within the genome are optimized and therefore, flexibility for choosing a target integration site is not necessary. For the purpose of fast and straight forward, marker free and scar less integration into production strains at a suitable site, protocols described above are laborious and slow and, in some cases, still need antibiotic based selection. Further, in silico design of guide RNAs and cloning steps are time consuming prerequisites in many integration protocols^7^. Here, we describe a new integration method that combines the λ-red system and the action of I-SceI in a different experimental set-up. In our integration system one single plasmid, the so called pAIO vector, is equipped with all features necessary for (1) target gene integration, (2) selection of positive clones and (3) subsequent plasmid self-curing without the need of antibiotic resistance markers. An engineered production strain contains the I-SceI recognition sequence at the target integration site. By successful insertion of the expression cassette, the I-SceI site is deleted. The gene encoding the enzyme I-SceI is present on the plasmid and, upon induction, cleaves genomic DNA. In case the recombination event did not occur properly, the bacterial host is killed. The high efficiency of this selection step keeps the screening effort to a minimum, and positive clones are identified fast and easily. In the last step, I-SceI cures cells from the helper plasmid by cleaving another I-SceI recognition site, which is present on the plasmid. Because of multiple copies of this vector, curing occurs after the selection step. Once, production strains are equipped with the I-SceI restriction site and the pAIO vector, a ready-to-use strain devoid of antibiotic resistance marker, genetic scares and helper plasmid can be established extremely fast and in just 4 steps.

## Results

### Generation of *E. coli* strains containing one I-SceI restriction site

**Figure 1:**
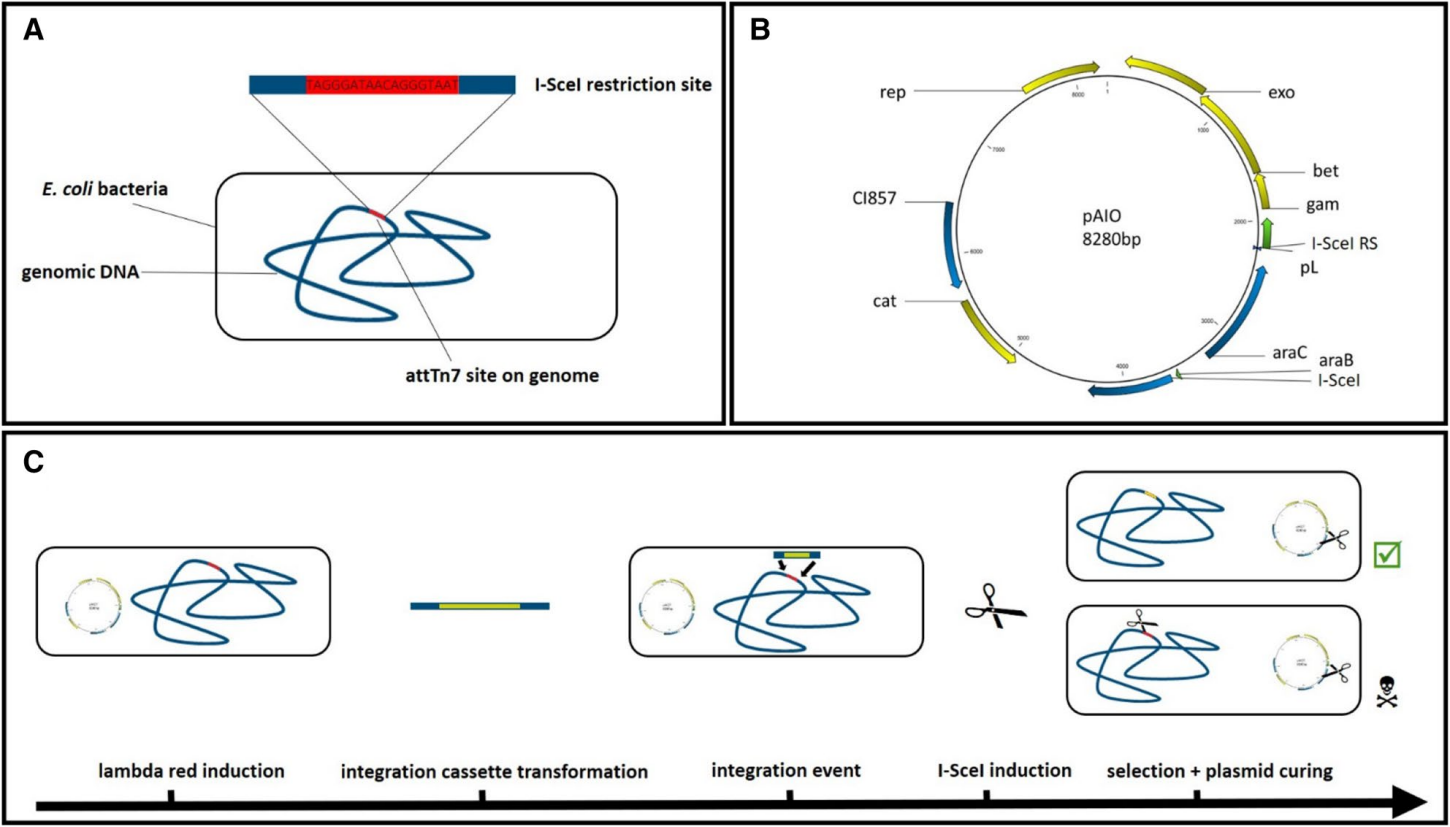
Schematic overview of the “All-In-One” genome integration method. (**A**) *E.coli*::I-SceIRS strain with I-SceI restriction site (RS) at the AttTn7-site on bacterial genome. (**B**) pAIO plasmid with ʎ-red genes (exo, bet, gam), arabinose inducible I-SceI, a chloramphenicol resistance gene (cat) and the I-SceI restriction site for plasmid curing. (**C**) Workflow of pAIO integration.

The recognition site of the I-SceI homing endonuclease was integrated into the genome of *E. coli* strains BL21(DE3) and HMS174(DE3) according to the method of Sharan et al.^9^. The 18 base long nucleotide sequence was inserted at the attTn7 site in combination with the kanamycin resistance gene. For identification of positive integrants, the genomic attTn7 site was amplified by PCR and subsequently digested in vitro with purchased I-SceI endonuclease. Sequencing further confirmed correct implementation of the I-SceI restriction site. Finally, the resistance gene was removed by the enzyme flippase. According to PCR and sequence analysis, resulting strains only contained the additional 18 bp comprising the I-SceI recognition sequence at the attTn7 site. Bacterial clones were designated BL21(DE3)::I-SceIRS and HMS174(DE3)::I-SceIRS (*E. coli*::I-SceIRS in Fig. 1A).

### Design of “All-In-One” integration vector pAIO

In order to create a plasmid carrying all features for “All-In-One” genome integration, we constructed the pAIO vector, which was equipped with following features. Heat inducible λ-red enzymes Exo, Gam and Bet, as described by Datta et al.^24^ were selected to ensure the integration of a linear target gene cassette into the *E. coli* genome. At 42 °C the conformation of λ-promoter repressor CI857, which was also encoded on the vector, is altered in a way that it releases the λ-promoter and enables transcription of the three recombination enzymes. For selection and plasmid curing, the gene encoding the homing endonuclease I-SceI was inserted into the vector under control of the araB promoter. Upon induction by arabinose, I-SceI is being produced and cleaves the genome of non-integrants thereby killing these cells. To provide accelerated plasmid curing, we engineered an additional I-SceI site into the plasmid itself, with the idea that the plasmid also gets cleaved upon the expression of I-SceI. Because of the multiple copies of the pAIO-vector and the relatively low expression of I-SceI, we assumed a delay in complete plasmid loss. Thereby, and by the fact that exo, gam and bet are being transcribed before induction with arabinose, sufficient amount of recombination enzymes remain in the cell in order to provide efficient integration and selection before plasmid curing. Further, the vector carried a constitutively expressed chloramphenicol resistance gene for selection and the pBBR1 origin of replication (Fig. 1B). The whole procedure of the “All-In-One” integration system is shown in Fig. 1C.

### Testing the killing effect of I-SceI

**Figure 2:**
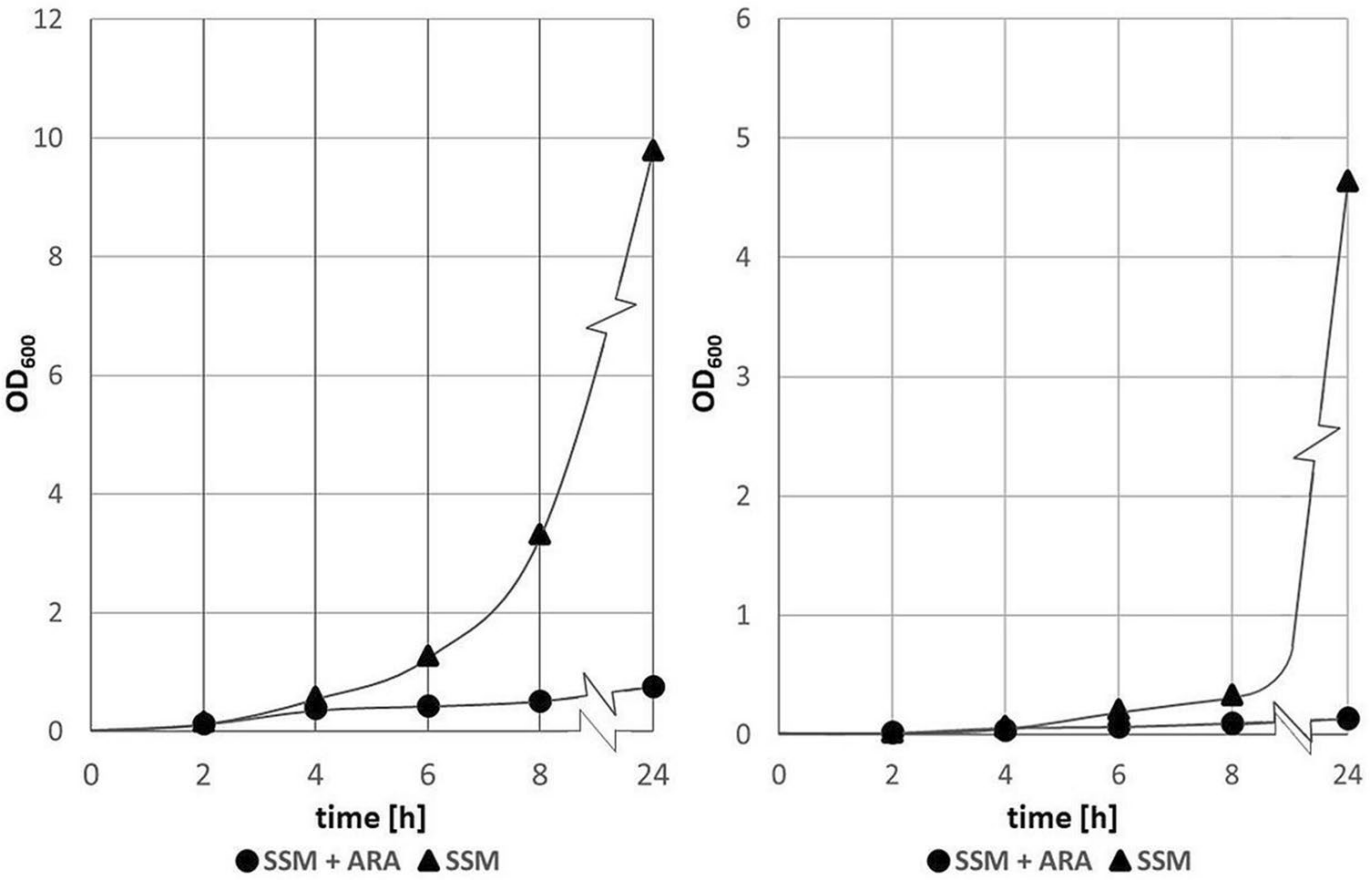
Measurment of OD_600_ with and without I-SceI induction at 5 different timepoints. Bacterial growth of BL21(DE3)::I-SceIRS + pAIO (**A**) and HMS174(DE3)::I-SceIRS + pAIO (**B**) in semi synthetic medium with and without arabinose.

The first evaluation step of our integration method was to test and confirm the killing effect of I-SceI encoded by the plasmid pAIO in *E. coli* BL21(DE3)::I-SceIRS and HMS174(DE3)::I-SceIRS cells. Therefore, the pAIO vector was transferred into each of the two engineered hosts and both strains were grown overnight. The next day, strains were diluted 1:100 in semi synthetic media supplied with glycerol instead of glucose and chloramphenicol (SSM + CM). In shake flasks cells were grown with and without 0.4 M arabinose. The optical density (OD_600_) of these induced and non-induced strains was determined at 5 timepoints. As shown in Fig. 2 induction of I-SceI led to a significant reduction of cell growth.

**Figure 3:**
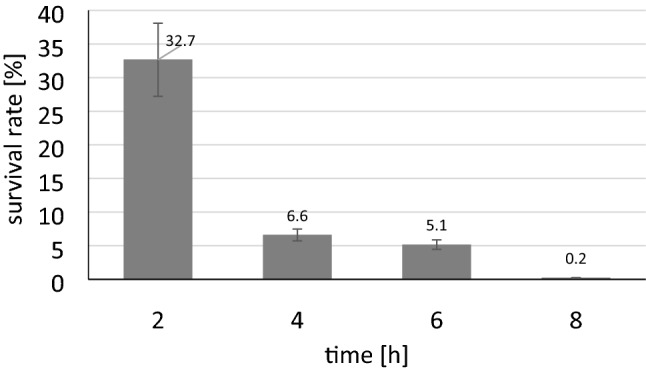
Survival rate of HMS174(DE3)::I-SceIRS cells after I-SceI induction. Cells were grown in SSM with and without arabinose and plated after 2, 4, 6 and 8 h onto LB agar plates supplied with chloramphenicol. Counted colonies, grown after I-SceI induction are given as percentage of colonies grown without I-SceI induction.

In a next experiment, we determined the survival rates on agar plates. Therefore, we plated I-SceI induced and non-induced samples of both strains on lysogeny broth agar with chloramphenicol (LB-CM) in different dilutions and counted the number of colony forming units. Figure 3 shows the proportion of surviving I-SceI-induced HMS174(DE3) cells in comparison to non-induced HMS174(DE3) cells. After 8 h, only 0.2% of the cells survived, indicating a non-complete but strong killing effect of I-SceI. In case of BL21(DE3)::I-SceIRS this effect was even stronger and faster (1% cells surviving after 2 h) than for HMS174(DE3)::I-SceIRS (data not shown). Results clearly confirmed that I-SceI expression effectively led to a double strand brake in the genome and thus, would be a suitable candidate for selection of an integration event without the requirement of an additional marker gene. Although, a 100% discrimination between positive and negative clones could not be achieved, the screening effort was assumed to be minimal.

### Screening selection efficiency of the pAIO system

**Figure 4:**
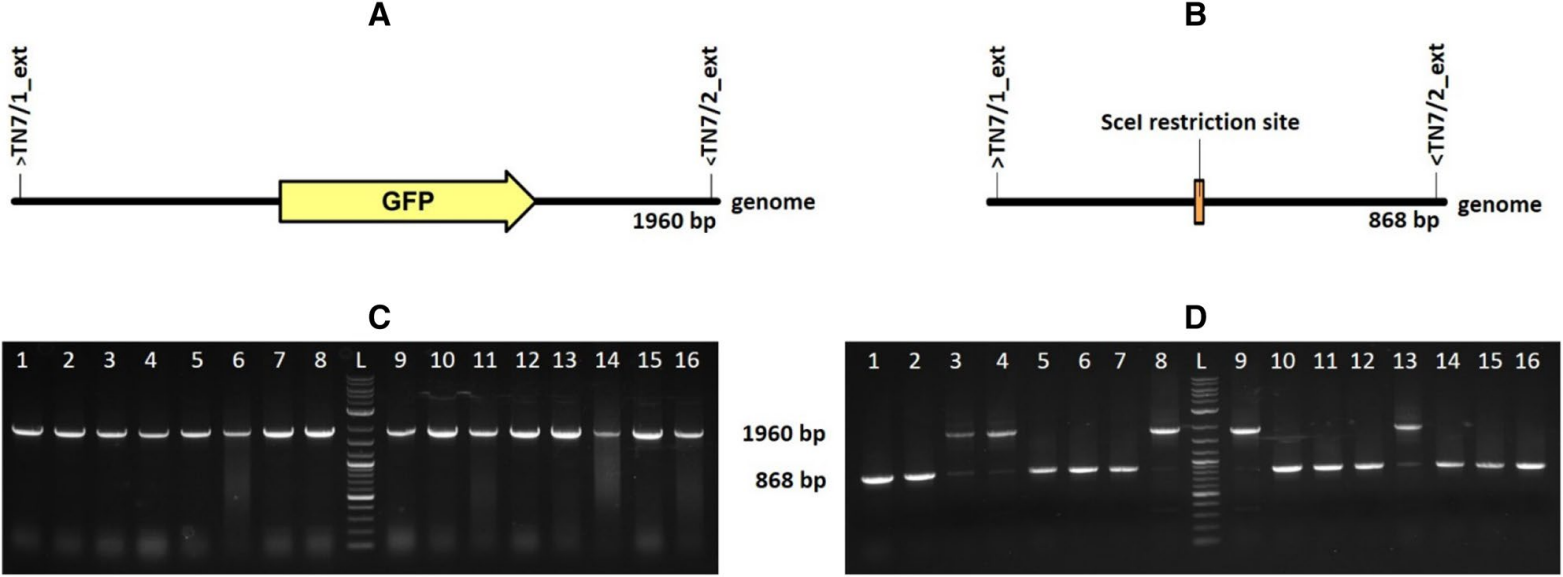
Colony PCR after GFP genome integration according to pAIO protocol. (**A**) Expected PCR product (1960 bp) in case of successful GFP integration. (**B**) Expected PCR product (868 bp) of empty genomic locus in case of failed genome integration. (**C**) Colony screening of 16 single BL21(DE3) < GFP > colonies. (**D**) Colony screening of 16 single HMS174(DE3) < GFP > colonies whereas colonies 3, 4, 8, 9, 13 were confirmed as positive integrants. (L…NEB 1 kb plus ladder).

In order to test integration, selection efficiency and required screening effort using the pAIO vector, genome integration at the attTN7 site was performed with green fluorescent protein (GFP) as the gene of interest (GOI). Therefore, an integration cassette consisting of GFP under control of the T7 promoter, was amplified by PCR using primers with 50 nucleotide overhangs (TN7_HO1, TN7_HO2) for homologous recombination in sense direction (in the leading strand of the origin of replication OriC). Genome integration was performed according to the protocol of Sharan et al. For selection of positive integrants, cells were diluted the next day in semi synthetic medium supplied with glycerol instead of glucose and 0.4 M arabinose for I-SceI induction. In case of BL21(DE3), cells were incubated for 4 h whereas HMS174(DE3) cells were incubated for 8 h. Then, cells were plated onto LB-agar plates supplied with chloramphenicol and arabinose. Positive integrants were identified by colony PCR amplifying the genomic attTn7 site. In each of 6 consecutively performed integration experiments, at least one positive clone could be detected among 16 screened colonies. This selection efficiency is considered to be high enough to enable fast and easy identification of positive integrants with a low screening effort. A representative screening result is shown in Fig. 4.

### Curing of pAIO

For plasmid curing after successful genome integration, a positive clone was grown in semi synthetic medium supplied with glycerol instead of glucose and 0.4 M arabinose, overnight at 30 °C. The next day, cells were diluted 1:100 in fresh medium and cultivated for further 8 h. Then, cells were transferred to LB-agar plates in a dilution that single colonies could be obtained. Cells were again incubated at 30 °C overnight. The next day 50 single colonies were picked and spotted onto LB-agar and agar supplied with chloramphenicol. After over-night incubation at 37 °C cured colonies could be identified. Whereas cells still carrying the pAIO vector grew on both agar plates, LB and LB supplied with chloramphenicol, cured colonies were only able to grow on antibiotic-free agar. Among the 50 colonies at least one completely cured genome-integrated colony was found in every pAIO genome integration experiment.

### Establishment of the “All-In-One” integration protocol

**Figure 5:**
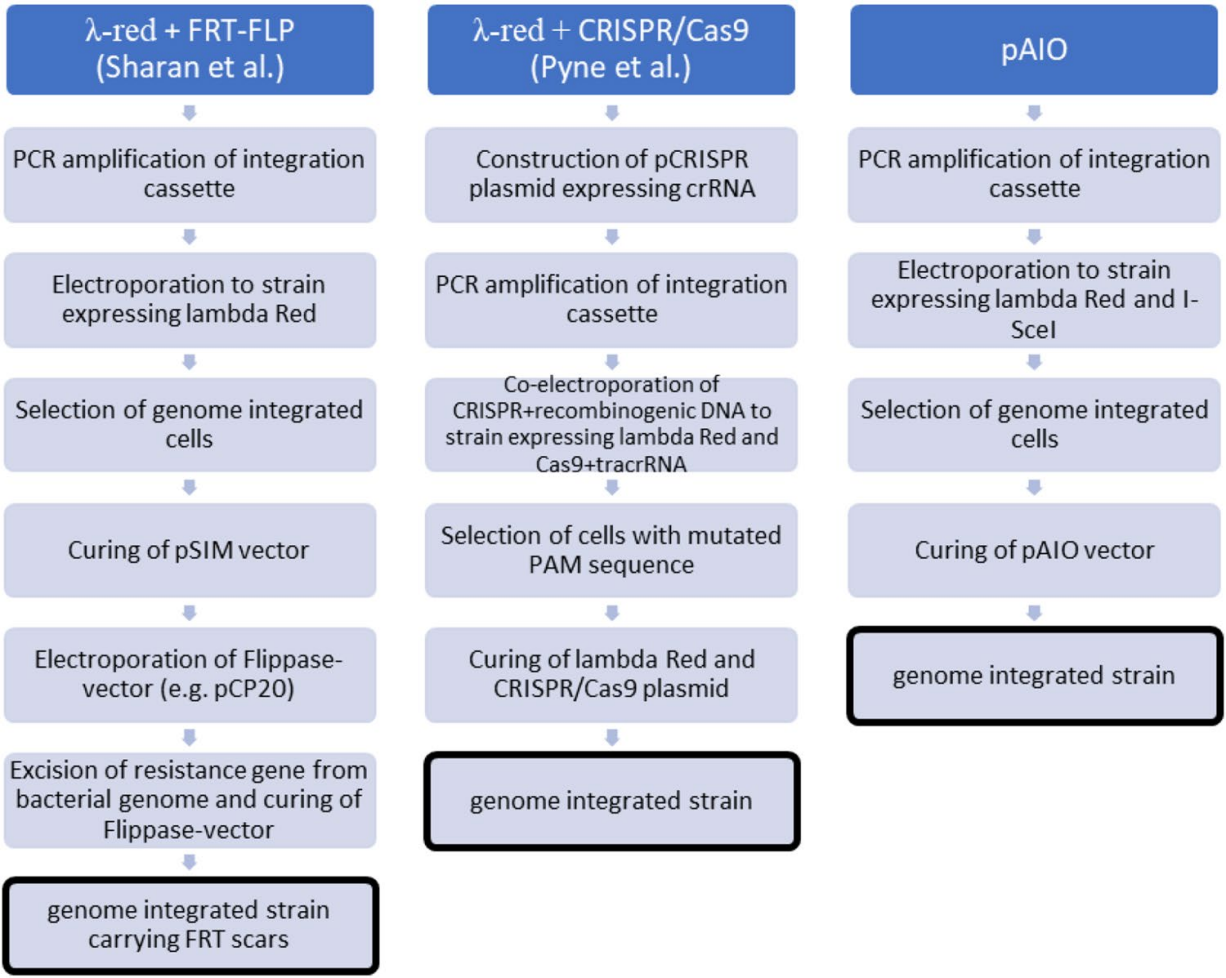
Schematic workflow of pAIO integration protocol and comparison to other integration methods. Genome integration according to the protocol of Sharan et al. (left), the method of Pyne et al. (middle) and the pAIO system (right) are compared.

Based on the previously described experiments a fast and straight forward protocol was developed. Starting from an engineered production strain harboring the pAIO plasmid, the required steps were (1) the amplification of the integration cassette by PCR, (2) electroporation of the PCR product into the pAIO harboring strain, (3) selection of positive clones by I-SceI induction via the addition of arabinose, whereby non-integrants still containing the intact I-SceI restriction site were killed by a double strand break in their genomes and (4) pAIO curing due to ongoing I-SceI endonuclease expression. The workflow of the protocol and a comparison to other integration systems is shown in Fig. 5, whereas details can be found in the “Materials and methods” part.

### Genome integration of various target genes

In order to further evaluate the established protocol, a variety of integration cassettes differing in nucleotide sequence and length were tested for efficient genome integration. Both engineered strains, BL21(DE3)::I-SceIRS and HMS174(DE3)::I-SceIRS were used for integration experiments. Target genes listed in Table 1 were integrated according to the pAIO protocol.

**Table 1:** Genes and gene cassettes that were integrated according to pAIO protocol.

Target protein	Description of target protein	Cassette length (bp)	Integration strain	Number of positive integrants out of 16 screened colonies
GFPmut3.1	Model protein	1367	B::I-SceIRS + pAIO	16
GFPmut3.1-KanR	Model protein with kanamycin resistance protein	2748	B::I-SceIRS + pAIO	14
ß-Lactamase	Ampicillin resistance protein	1392	B::I-SceIRS + pAIO	16
6His-mCherry-dsbA^SS^sfGFP	Combination of cytoplasmic (mCherry) and periplasmic model protein (sfGFP)	1890	B::I-SceIRS + pAIO	10
dsbA^SS^sfGFP	Periplasmic model protein	1100	B::I-SceIRS + pAIO	8
6His-mCherry	Cytoplasmic model protein	1087	B::I-SceIRS + pAIO	10
ompA^SS^TNFα	Tumor necrose factor	2489	B::I-SceIRS + pAIO	14
6His-BIWA4 (scFv)	Human single chain antibody fragment targeting cell-surface glycoprotein	900	B::I-SceIRS + pAIO	6
6His-hFGF-2	Fibroblast growth factor	2801	B::I-SceIRS + pAIO	5
ompA^SS^6His-mTNFα	Tumor necrose factor with His-tag	2507	B::I-SceIRS + pAIO	12
Fab: dsbA^SS^-BIBH1	Human antibody fragment targeting fibroblast Activation protein with N-terminal dsbA-leader	2208	B::I-SceIRS + pAIO H::I-SceIRS + pAIO	4 2
Fab: ompA^SS^BIBH1	Human antibody fragment targeting fibroblast Activation protein with N-terminal ompA-leader	2208	B::I-SceIRS + pAIO H::I-SceIRS + pAIO	6 4
Fab: dsbA^SS^BIWA4	Human antibody fragment targeting cell-surface glycoprotein with N-terminal dsbA-leader	2157	B::I-SceIRS + pAIO H::I-SceIRS + pAIO	9 1
Fab: ompA^SS^BIWA4	Human antibody fragment targeting cell-surface glycoprotein with N-terminal ompA-leader	2157	B::I-SceIRS + pAIO H::I-SceIRS + pAIO	10 2
Fab: dsbA^SS^FTN2	Human antibody fragment targeting tumor necrose factor with N-terminal dsbA-leader	2172	B::I-SceIRS + pAIO H::I-SceIRS + pAIO	2 3
Fab: ompA^SS^FTN2	Human antibody fragment targeting tumor necrose factor with N-terminal ompA-leader	2172	B::I-SceIRS + pAIO H::I-SceIRS + pAIO	11 2

sfGFP…superfolder GFP, 6His…N-terminal His-tag, dsbA^SS^…signal peptide of periplasmic thiol:disulfide interchange protein DsbA, ompA^SS^…signal peptide of periplasmic outer membrane protein A, B… BL21(DE3), H…HMS174(DE3).

## Materials and methods

### Strains, media and cultivation

For cloning purposes, chemically competent *E. coli* NEB-5α cells were purchased from New England Biolabs (NEB, Ipswitch, USA). For genome integration and I-SceI expression, *E. coli* BL21(DE3) (NEB) and HMS174(DE3) (Novagen, Madison, USA) were used. Cells were routinely cultured in Lysogeny Broth (LB) media, recovered in super optimal broth medium supplemented with 20 mM glucose (SOC media) and plated on LB agar. The following antibiotic concentrations were used: ampicillin (Amp) 100 µg/mL, chloramphenicol (Cm) 20 µg/mL. Shake flask cultivations were conducted in in semi synthetic medium with glycerol as sole carbon source at 37 °C. In the presence of the pAIO vector the temperature was set to 30 °C. Overnight cultures were grown in LB-medium supplied with chloramphenicol (20 µg/mL). In case of I-SceI induction, arabinose (0.4 M) was added to the culture.

### PCR primers

Primers were ordered from Sigma-Aldrich (St. Louis, USA) and Integrated DNA Technologies (IDT, Coralville, USA). For PCR, primers shown in Table 2 were diluted to a concentration of 10 pmol/µL.

**Table 2:** Primer names and sequences.

TN7_HO1	AGATGACGGTTTGTCACATGGAGTTGGCAGGATGTTTGATTAAAAACATA GTAGTAGGTTGAGGCCGTTG
TN7_HO2	CAGCCGCGTAACCTGGCAAAATCGGTTACGGTTGAGTAATAAATGGATGC CGGATATAGTTCCTCCTTTCAG
EcoRI_pROCOLI_sense	GGAGGAATTCACCATGGTACCCGG
NheI_pROCOLI_as	CTCCTGCTAGCCCAAAAAAACGGG
FRT_cm_sense	AGATGACGGTTTGTCACATGGAGTTGGCAGGATGTTTGATTAAAAACATAtagggataacagggtaatTAAGCAGAAGGCCATCCTGAC
FRT_cm_as	CAGCCGCGTAACCTGGCAAAATCGGTTACGGTTGAGtaaTAAATGGATGCGCGAACGTGGCGAGAAAGG
TN7/2_GFP	CAGCCGCGTAACCTGGCAAAATCGGTTACGGTTGAGTAATAAATGGATGCATTTAGAGCTTGACGGGG
SceIRS_sense	tagggataacagggtaatCGTAGAGGATCTGCTCATG
SceIRS_as	TCTCACCTACCAAACAATGC
TN7/1_ext	ACCGGCGCAGGGAAGG
TN7/2_ext	TGGCGCTAATTGATGCCG
BglII_SceI_sense	atcatcagatctGATGCGTCCGGCGTAGAGG
BglII_SceI_as	atcatcagatctTTCGCGGCCGCTGTATTTAG

### Electroporation

Chemically competent Neb 5 alpha cells were bought from New England Biolabs and transformed according to the manufacturer’s protocol. Electrocompetent cells were prepared as described by Sharan et al.^9^. Electroporation was performed in 1 mm gap cuvettes using 50 µL of competent cells. The following settings were chosen: 1800 V, 25 µF, 200 Ω. After electroporation cells were recovered in SOC-medium.

### Construction of pAIO

The I-SceI gene was ordered from Integrated DNA Technologies (IDT, Coralville, USA) as a gene block containing restriction sites NheI (5′) and EcoRI (3′). Via these sites I-SceI was cloned into the pROCOLI vector downstream of the arabinose inducible pBAD promoter. This plasmid is a derivative of vector plkJ12^25^ where the ampicillin resistance marker was changed to FRT-flanked chloramphenicol resistance gene. From the pROCOLI vector the pBAD-I-SceI fragment was amplified by PCR using primers BglII_SceI_sense and BglII_SceI_as. Subsequently the fragment was cloned into pSIM7 via BglII restriction sites, resulting in plasmid pAIO. For insertion of the I-SceI restriction site (I-SceIRS) into the vector backbone, a PCR was performed using primers I-SceIRS-sense (carried the I-SceI restriction site as a primer overhang) and I-SceIRS-as and pAIO as template.

### Integration of the I-SceI recognition site into the bacterial chromosome

For the amplification of the I-SceIRS-FRT-cm-FRT integration cassette, primers FRT_cm_sense and FRT_cm_as were used and vector pROCOLI was taken as template. Integration at the attTn7 site was performed according to the protocol of Sharan et al.^9^. The antibiotic resistance marker was removed using the FLP-FRT system using FLP recombinase encoded on pCP20 vector^26^.

### Construction of integration cassettes

All target genes were amplified from vector pET30a by PCR, using primers TN7_HO1 and TN7_HO2, which bind to the vector backbone. Target genes were placed at the multiple cloning site inside the NdeI (NEB) and EcoRI (NEB) restriction sites. After amplification of integration cassettes, plasmid template DNA was digested with restriction enzyme DpnI (NEB) and gene cassettes were column-purified using Monarch PCR and DNA cleanup Kit (NEB), according to the manufacturers protocol.

### Genome integration according to “All-In-One” method

A general outline of this method is shown in Fig. 5.

Day 1: Vector pAIO is transferred into 50 µL of electrocompetent engineered *E. coli*. After electroporation cells are recovered in SOC medium at 30 °C shaking (700 rpm) for 1 h. Cells are plated onto LB-cm agar (20 µg/mL) and grown over night at 30 °C.

Day 2: A single colony is used for inoculation of an over-night shake flask culture in LB-cm (20 µg/mL) at 30 °C.

Day 3: 500 µL of over-night culture are used for inoculation of 35 mL of fresh LB-cm (20 µg/mL) medium. Cells are grown to an OD_600_ of 0.7–0.8 (approximately 2.5 h–3 h) at 30 °C. For λ-red induction 17 mL of culture are transferred to a 125 mL shake flask and put into the water bath at 42 °C for 15 min (induced cells), shaking. Immediately after 15 min. cells are chilled in an ice water slurry and shaked gently. Then, cells are made electrocompetent by washing with ice cold water as follows:

1. Pellet cells in 50 mL falcon tubes at 4600 g for 7 min.
2. Wash with 30 mL of ice-cold water.
3. Resuspend cells in 1 mL of ice-cold water and transfer into 1.5 mL Eppendorf tubes. Centrifuge at 10,000g for 30 s and aspirate the supernatant.
4. Repeat step 3.
5. Resuspend cells in 200 µL of ice-cold water.

5 µL of integration cassette (500 ng–1 µg) are added to 50 µL of competent cells. Electroporation is performed in a 0.1 mm cuvette using the following settings: 1800 V, 25 µF, 200 Ω.

Cells are recovered in 950 µL of LB-cm (20 µg/mL) medium and integration is performed over night at 30 °C shaking.

Day 4: Cells are pelleted for 2 min at 4600 g and resuspended in 1 mL of SSM + glycerin. Cells are diluted 1:100 and 1:10 in 1 mL of fresh SSM + glycerin supplied with 0.4 M arabinose. After growth for 4 h (in case of HMS(DE3), for 8 h!) at 30 °C shaking, cells are plated onto LB-cm (20 µg/mL) + arabinose (0.4 M) agar (undiluted, 1:10 dilution).

Day 5: Colonies are screened by PCR using TN7ext-primers. Master plates are prepared on LB and LB-cm (20 µg/mL) + arabinose (0.4 M) agar plates.

Day 6: 10 mL of SSM + glycerin supplied with 0.4 M arabinose are inoculated with selected colony from LB-master plate. Cells are grown over night at 30 °C shaking.

Day 7: The over-night culture is diluted 1:100 with fresh SSM + glycerin supplied with 0.4 M arabinose and grown at 30 °C, shaking. After 8 h cells are stroked out in a way that single colonies can be obtained.

Day 8: 50 colonies are picked and resuspended in 10 µL water. 2 µL are spotted onto LB and LB-cm (20 µg/mL) agar plates.

Day 9: Identification of plasmid cured cells: growth on LB but not on LB-cm (agar and liquid medium).

## Discussion

Many studies in the past^4^ have shown that genome integration of a gene of interest is preferable for recombinant protein expression in many industrial applications. In contrast to plasmid-based expression, maintenance of the target gene throughout the production process is guaranteed and the metabolic load derived from other plasmid encoded components is abolished. *E. coli* strains HMS174 and BL21 constitute the most common industrial production platforms for biopharmaceuticals. Although, gene integration methods for these strains are available, there was a need for improvement in terms of time, robustness and effort that was required. In this work, a genome integration method was developed that requires only one single helper plasmid. The so called “All-in-One” plasmid pAIO carries all features necessary for successful integration at the *E. coli* attTn7 site. Most other integration methods are based on simultaneous or sequential transfection of at least two plasmids^7^. Unlike other helper plasmids which often carry a temperature sensitive ori, the pAIO vector is cured by self-cleavage. Thereby thermal damage and heat derived negative influence on plasmid replication causing plasmid loss is prevented throughout the whole integration procedure. McKenzie et al.^27^, as well, describe a genome integration method that is based on a single plasmid. However, in this integration method a cloning step of the gene of interest into a 12,549 bp vector is required. Such big vectors are often fragile, are inefficient in transfections and might get destroyed during the cloning procedure. While this method depends on Tn7-transposase enzymes, whereby integration is possible solely at the attTn7 site, this work includes the possibility to position the I-SceI restriction site at any desired genomic locus. Zhao et al.^28^ describes a one-plasmid method where the lambda-red system is combined with CRISPR/Cas9. However, construction of this plasmid involves in silico design of guide RNA as well as vector assembling steps. Whenever the genomic integration site is changed the whole vector needs to be redesigned. In the “All-In-One” system” integration vector pAIO as well as the pAIO protocol can still be used without any adaptions although another genomic site is engineered for integration. The relatively long restriction site of I-SceI makes it unique throughout the bacterial genome. Thus, off target effects as well as the need for PAM near the target integration site, don’t need to be considered. Unlike in systems using CRISPR/Cas9 for selection, no in silico design of RNAs, co-expression of RNAs or cloning steps need to be performed in advance. Once the production strain has been equipped with the I-SceI restriction site and the pAIO vector, a linear, PCR-amplified integration cassette is the only prerequisite to get the protocol started.

Since the target site of integration is often fixed in industrial production processes, the requirement for flexible integration was dismissed. Individual testing of various positions within a genome for optimal protein expression is laborious and the benefits have yet to be proven. Best practice is to choose a position that has shown stable and efficient gene expression for several candidates in one host. The attTn7-site is used in many standard production strains and has been proven optimal for *E. coli* strains HMS174(DE3) and BL21(DE3) in industrial applications. Whenever another optimal integration site for a specific *E. coli* strain is identified, the I-SceI site can easily be inserted at any position wanted.

Simple handling and short time consumption make the “All-In-One” method superior to commonly used standard procedures. The reduced number of working steps minimizes potential sources of error and make the “All-In-One” system very robust. With a screening effort of only 16 colonies, we consider the efficiency of the pAIO system as highly sufficient for fast and easy use in the lab. Especially, when a large number of different strains has to be generated or if genes have to be tested rapidly, we consider the pAIO based strategy as beneficial for biopharmaceutical clone production and industrial bioprocessing.
